# Bibliometric and Co-Occurrence Study of Process System Engineering (PSE) Applied to the Polyvinyl Chloride (PVC) Production

**DOI:** 10.3390/ma16216932

**Published:** 2023-10-28

**Authors:** Ángel Darío González-Delgado, Miguel Ramos-Olmos, Nórida Pájaro-Gómez

**Affiliations:** 1 Nanomaterials and Computer Aided Process Engineering Research Group (NIPAC), Chemical Engineering Department, Universidad de Cartagena, Cartagena 130015, Bolívar, Colombia or miguel.ramos.o@uniminuto.edu (M.R.-O.); npajarog@unicartagena.edu.co (N.P.-G.); 2 Grupo de Investigación en Ciencias Administrativas y Seguridad y Salud en el Trabajo (CIASST), Business Administration Department, Universidad Minuto de Dios-UniMinuto, Cartagena 130001, Bolívar, Colombia

**Keywords:** PVC production, process simulation, co-occurrence analysis, cluster analysis

## Abstract

PVC is widely used in packaging, electrical insulation, and medical devices due to its versatility owing to its resistance, incombustible and barrier properties as well as affordable cost. In the present study, bibliometric and co-occurrence analyses are proposed to identify trends, gaps, future directions, and challenges regarding process system engineering (PSE) applied to the production process of PVC using VOSviewer as a tool for analyzing the data obtained from SCOPUS. A mapping of different topics alluding to simulation of PVC production was provided to gain a better insight into the development of the topic and its progression. The findings indicate that the literature on this topic falls into five different clusters: modeling and simulation of PVC production, process control and optimization, and optimization strategies of the process. From a co-occurrence study we identified that mathematics and statistics applied to polymer chemistry, separation phenomena, and polymer production are the main areas of interest for further research. The trends suggest that Monte Carlo and numerical simulation can contribute to a deeper understanding of PVC’s properties and behavior. In addition, the focus on plastics and microplastics reflects concerns about the environmental impact. A bibliometric study evidenced that PSE provides the tools for improvement in PVC production processes by employing advanced process engineering techniques. Modelling and new algorithms for simulation methods of continuous polymerization processes are important to enhance accuracy and efficiency across various applications. The study also proposes a research agenda for future researchers working in the field of the use of PSE applied to the PVC production process.

## 1. Introduction

PVC stands for polyvinyl chloride, which is a synthetic thermoplastic polymer [1]. PVC is commonly used in construction, packaging, electrical insulation, clothing, and medical devices due to its versatility, durability, resistance to weather conditions, low flammability, excellent barrier properties, eco-friendliness, and low cost, establishing a strong presence of PVC-based products in the global market [2]. It is estimated that PVC is the third most produced plastic in the world, after polyethylene and polypropylene [3,4]. PVC production involves the polymerization of vinyl chloride monomer (VCM) to form PVC resin [5]. VCM is typically produced through the chlorination of ethylene in the presence of a catalyst. The process involves several steps, including ethylene chlorination, cracking, and separation [6].

There are three main PVC production processes: suspension, emulsion, and bulk polymerization [7]. In suspension polymerization, VCM is dispersed in water with the help of a suspending agent and initiator and polymerized to form PVC particles. These particles are then separated from the reaction mixture and washed to remove any unreacted monomer and impurities [8]. In emulsion polymerization, VCM is dispersed in water with the help of a surfactant and polymerized to form PVC particles. The resulting emulsion is stabilized with the help of a protective colloid, and the PVC particles are then coagulated and washed to remove any unreacted monomer and impurities [9]. In bulk polymerization, VCM is polymerized directly without any solvent or dispersing agents. The polymerization takes place in a closed vessel with the help of initiators and under heat and pressure. Bulk polymerization produces PVC with a low molecular weight and a relatively low residual monomer content. This method is less common than the other two methods due to challenges in handling the heat generated during polymerization and controlling the molecular weight distribution [10].

Several parameters affect the properties of PVC, influencing its performance, processability, and end-use applications; some of the key parameters include molecular weight and molecular weight distribution, the degree of polymerization, residual monomer content, polymerization method, processing conditions, as well as the degree of branching or use of additives such as plasticizers, stabilizers, and pigments to enhance its properties and performance. For example, it has been found that PVC encounters less resistance when combined with certain natural fibers [11]. Compounding is typically performed using high-speed mixers or extruders [12]. After compounding, PVC can be processed using techniques such as extrusion, injection molding, and calendaring to produce finished products such as pipes, profiles, sheets, and films [13]. PVC production is a complex process that involves a range of chemical reactions, polymerization techniques, and processing methods, and requires careful control and monitoring to ensure consistent quality and performance of the final product.

Process systems engineering (PSE) is a multidisciplinary field that integrates principles from chemical engineering, computer science, mathematics, and control theory to optimize the design, operation, and control of chemical processes [14]. PSE includes process modeling and simulation, which involves the development of mathematical models that describe the behavior of chemical processes [15]. These models can be used to simulate the behavior of processes under different conditions and to optimize process design and operation [16]. Process optimization involves the use of mathematical models and optimization techniques to identify the optimal operating conditions for a process. Optimization can be used to minimize production costs, maximize product quality, minimize energy consumption, and reduce environmental impacts [17]. Process control involves the use of control systems to maintain process variables within desired ranges and to ensure the safe and efficient operation of chemical processes. Control systems can be implemented using a variety of techniques, including feedback control, feedforward control, and model predictive control [18]. Process integration involves the design of integrated process systems that minimize energy consumption and environmental impacts. This can be achieved using heat or mass exchangers, process integration networks, and pinch analysis [19].

Process systems engineering plays a crucial role in the production of polyvinyl chloride (PVC) because PSE techniques are employed to optimize the various chemical and physical processes involved in PVC production; the optimization can be performed by applying integration techniques for improving overall efficiency, reducing energy losses, and minimizing the environmental footprint, resulting in efficient resource utilization [20]. This includes reaction kinetics, heat and mass transfer, and process control. Optimization ensures that the production processes are carried out at their maximum efficiency, leading to higher yields and reduced energy consumption. In addition, the design of a safer and more sustainable process involves risk assessment, hazard identification, the development of safety protocols to minimize accidents, chemical spills and other safety hazards [21,22], complying with environmental regulations, as well as minimizing the release of pollutants [23]. Furthermore, PSE encompasses advanced process control and automation systems [24,25] and data analysis and modeling to make informed decisions [26]. In summary, PSE is integral to PVC production, aiding in the process design and optimization, ensuring sustainable operation, assessing the environmental impact, controlling energy consumption and emissions, managing the use of additives, and guaranteeing product quality.

PSE is extremely important for improving the efficiency, sustainability, and profitability of PVC manufacturing by addressing various aspects of the production process, process design and optimization. PSE facilitates the design and optimization of PVC production processes, resulting in energy-efficient and cost-effective operations, and allowing engineers to identify optimal operating conditions and process configurations. With respect to process control and monitoring, PSE can contribute to the development of advanced process control strategies, ensuring the stability and safety of PVC manufacturing processes, and can enhance the control and monitoring of critical process variables. In addition, PSE promotes the integration of sustainability principles into PVC manufacturing processes considering the environmental, economic, and social aspects of the production chain. Life cycle assessment (LCA) and eco-efficiency analysis are examples of PSE techniques used to evaluate the environmental impact of PVC or plasticizers for PVC applications, enabling the identification of areas for improvement and the development of eco-friendly manufacturing practices [27]. Regarding energetic transition and decarbonization, PSE can facilitate the integration of renewable resources into PVC production processes, reducing dependency on fossil fuels and minimizing greenhouse gas emissions.

## 2. PVC Production Process Alternatives

Stages for the production of PVC depend on the technology used for polymerization and purification, but in general terms, the process involves the steps of production of VCM, which if typically achieved by the reaction of ethylene and chlorine, polymerization, where the VCM is then converted into PVC, isolation and drying, where PVC particles are separated from the water suspension or emulsion by methods such as filtration or centrifugation, means that the separated PVC particles are then washed to remove any remaining impurities and dried to obtain the final PVC powder. There can be additional stages such as compounding or processing and shaping, as stated earlier. Production of PVC can be made using several routes such as suspension, emulsion, and bulk [28].

### 2.1. Suspension Polymerization

The suspension polymerization method is a common method used to produce PVC resin, involving the stages of preparation of raw materials [29]. The stabilization of VCM droplets in water, polymerization, and recovery of the resulting PVC particles. A common formulation for PVC production consists of 180 parts water, 100 parts of VCM, and minor quantities of dispersants (less than one part), initiator soluble in monomer, and chain transfer agents such as trichloroethylene [30]. The VCM is typically obtained by the chlorination of ethylene and the suspending agents and initiators are added to the water; in formation of the droplets stage, the VCM is added to the water containing the suspending agents and initiators and the suspending agents help to stabilize the droplets of VCM in the water, preventing them from coalescing [31]. In the polymerization stage, the initiators added to the water cause the VCM droplets to polymerize and the reaction is exothermic and generates heat, which is controlled through cooling. In the particle formation stage, the polymerization of VCM forms PVC particles suspended in the water and the size of the particles can be controlled by varying the reaction conditions, such as the number of initiators and suspending agents. Finally, in the recovery stage, the resulting PVC particles are separated from the water using centrifugation or filtration, washed to remove any residual chemicals, and dried. Producing PVC through suspension offers multiple benefits, such as a high PVC yield, effective control over particle size, and the capacity to create high molecular weight PVC [32].

Some efforts have been made to optimize the suspension polymerization process in order to improve the efficiency of PVC production. Dai, Zhang and Jin [33] examined the impact of the particle size on the molecular weight distribution of low polymerization degree PVC resins across three grades. They found a relationship between the number of sub-micro particles and both the molecular weight and molecular weight distribution. A higher number of sub-micro particles led to increased molecular weight and molecular weight distribution. Hohmann et al. [34] investigated solid/liquid suspension flow behavior in horizontal helical coil tubes, focusing on PVC beads/water. They identified three flow regimes and developed empirical correlations to define transitions between them. Their study found a narrow particle residence time distribution close to the ideal plug flow, assisting in equipment and process design. Georgiadou et al. [35] investigated the dispersion of inorganic nanoparticles such as calcium carbonate, silica, and hydrotalcite in VCM during suspension polymerization. This resulted in PVC grains with a higher porosity, a distinct internal morphology, and different size distribution compared to commercial PVC. However, the distribution of nanoparticles within PVC grains was not uniform, with regions of varying filler concentration and pure polymer. Tierno et al. [36] found that PVC resins produced using continuous initiator dosing (CiD) technology exhibited improved color and thermal stability compared to conventional PVC. Various tests were conducted to characterize thermal stability, while the color was evaluated using spectrophotometric measurements. The superior properties of CiD PVC resins could be attributed to a lower level of polymer chain defects and a reduced residual initiator amount, resulting in better color and thermal stability of the resin.

Park et al. [37] investigated the influence of dual impeller geometry on the droplet size in the suspension-PVC (S-PVC) polymerization process. They utilized 1,2-dichloroethane as a dispersed phase to simulate the process, as it is a less toxic alternative to VCM. The borescope method was employed to measure the droplet size in a biphasic liquid system, and the Sauter mean diameter increased by 46.5% when the upper paddle impeller was replaced by a 20° pitched paddle. The diameter also increased with larger impeller diameters and blade widths. The authors additionally revised a geometrical factor (F) based on these findings and used a calculated maximum energy dissipation rate to establish a correlation for the Sauter mean diameter. This proposed correlation can estimate the Sauter mean diameter with a ±20% error margin, enabling predictions of polymerization normality under specific impeller geometries. Zouhri et al. [38] explored the use of ascorbic acid derivatives combined with ferrocenyl-based activators in catalytic amounts as powerful redox systems for promoting the decomposition of organic peroxides into radicals. These radicals facilitate the polymerization of VCM under suspension conditions. The initial polymerization rates can be improved up to eightfold, depending on the vinylic monomer and peroxide. The use of a ferrocene/ascorbyl palmitate activator reduced the overall polymerization time of VCM from 3 to 2 h under industrial conditions. However, issues such as crusting during polymerization and yellowing of PVC upon thermal tests were observed. Yang et al. [39] developed a novel one-pot method for preparing PVC resins with enhanced properties by incorporating crosslinked polyacrylate latex with tertiary amine groups (ACLN) and base latex without tertiary amine groups (ACL) using emulsion and suspension polymerization. The study found that ACLN/PVC preparation avoided reactor scaling, displayed improved thermal stability, and exhibited increased toughness without affecting the tensile strength and softening temperature. These improvements were attributed to a covalent and ionic bond formation between ACLN and PVC, leading to superior PVC products for various applications. Guo et al. [32] investigated the effects of agitation speed on the conversion and morphology of PVC polymer grains. Their findings revealed that agitation has a significant impact on the size distribution of polymer particles, although it does not influence conversion rates.

### 2.2. Emulsion Polymerization

Emulsion polymerization is a significant process in PVC production, accounting for about 10% of all PVC manufactured. This method is distinct from the suspension process, mainly because it utilizes an anionic surfactant instead of a water-soluble polymeric suspending agent. Often, as much as 1.5% of the anionic surfactant is used in this process. The initiator employed is water-soluble, typically an inorganic peroxide requiring a trace of a metallic ion, such as Cu^+^. The kinetics of emulsion polymerization differ substantially from those of the suspension process [40]. PVC is synthesized by emulsifying VCM in water with the help of a surfactant, which stabilizes the resulting emulsion. The emulsion is then polymerized in the presence of initiators, which generate free radicals that initiate the polymerization process. The process can be divided into four main stages: initiation, propagation, termination, and coagulation. The initiation stage involves the generation of free radicals from initiators such as ammonium persulfate and sodium bisulfite, which can be enhanced by the addition of reducing agents such as ascorbic acid or sodium thiosulfate; these free radicals then react with VCM molecules to form PVC radicals, which undergo further polymerization through a process known as propagation. During propagation, the PVC chains grow longer as new VCM monomers are added to the growing polymer chain. The propagation stage can be affected by several factors, including the concentration of VCM and the presence of other monomers or additives [41]. The termination stage occurs when two PVC chains combine to form a PVC molecule, effectively stopping the polymerization process. Termination can occur through several mechanisms, including the combination of two growing chains, the disproportionation of a growing chain into a small and a large chain, or the termination by a chain transfer agent. The choice of initiator can also affect the termination mechanism and the molecular weight distribution of the resulting PVC [42]. Finally, the coagulation stage involves the separation of the PVC particles from the emulsion. Coagulation can be achieved through various methods, such as the addition of coagulants or a change in the pH, temperature, or ionic strength of the system. The choice of coagulation method can affect the particle size distribution and the quality of the final PVC product.

The finished resins produced by emulsion polymerization are utilized in a wide variety of specialty products and employ unique processing techniques. The terms “plastisol” or “paste” are often used to describe the resins of emulsion polymerization and the fabrication technologies that transform them into finished products. Rasteiro et al. [42] investigated the rheology of plastisols made from PVC samples produced by the emulsion process, focusing on the relationship between particle size distribution, surface characteristics, and plastisol aging, and revealed a significant influence of the original polymer properties on the viscosity aging and viscoelastic behavior of plastisols. A substantial decrease in viscosity can be achieved by altering a specific surfactant during polymerization. Furthermore, the type of particle aggregates present in the powder can determine the evolution of particle size in the plastisol, which directly correlates with observed changes in aging and viscoelastic behavior. Because the rheology of PVC plastisols is influenced by factors such as formulation components, concentration, temperature, and polymer properties, Tomás et al. [43] added an ester-type emulsifier in a post-polymerization stage, in addition to typical polymerization surfactants. The results demonstrated particle aggregation in the initial aqueous dispersion (latex), leading to a substantial reduction in viscosity and the aging profile, and altered viscoelastic properties in the resulting plastisols.

The emulsion polymerization of PVC is a complex process that is affected by several factors, including the choice of surfactant, initiator, and reaction conditions such as temperature and pH. For example, the choice of surfactant can have a significant impact on the size and morphology of the resulting PVC particles, which in turn can affect the mechanical properties of the final product. Similarly, the choice of initiator can affect the rate and efficiency of the polymerization process. Savrik et al. [41] studied the impact of emulsion process formulation ingredients on the morphology, structure, and properties of PVC powder, which was composed of spheres and exhibited a broad particle size range, spanning from 10 nm to 20 μm as observed through scanning electron microscopy (SEM). The specific surface area of the PVC powder was determined to be 16 m^2^/g from methylene blue adsorption at 25 °C and 12 m^2^/g from nitrogen adsorption at −196 °C. Atomic force microscopy (AFM) revealed that the surface roughness of the films obtained by pressing the particles was 25.9 nm. FTIR spectroscopy indicated that the PVC powder contained carbonyl and carboxylate groups belonging to additives, such as surface-active agents, plasticizers, and antioxidants used in PVC production. The mass of these additives was found to be 1.6% of the PVC powder, as determined by ethanol extraction. Energy-dispersive X-ray (EDX) analysis showed that the surfaces of the PVC particles were coated with carbon-rich materials. These coatings exhibited a plasticizing effect, as the glass transition temperature for the PVC powder was lower than 25 °C, while it was 80 °C for the ethanol-extracted powders, as determined by differential scanning calorimetry. These additives, which originated from the polymerization process, made the PVC powder more thermally stable, as evidenced by Metrom PVC thermomat tests.

Recent studies on the emulsion polymerization of PVC have focused on developing new initiators, optimizing reaction conditions, and improving the properties of the final product through mixtures of MVC with other monomers or nanoparticles. The study of Niu et al. [44] introduces a straightforward method to create anisotropic P(VC-co-AAEM)/PS nanoparticles with adjustable shapes using emulsifier-free seeded emulsion polymerization. First, non-cross-linked P(VC-co-AAEM) seeds with a hydrophilic surface are synthesized by copolymerizing VCM and acetoacetoxyethyl methacrylate (AAEM). These seeds are then employed to produce P(VC-co-AAEM)/PS nanoparticles with numerous bulges through styrene emulsion polymerization. Electron microscopy analysis revealed that the amount of AAEM in the seeds is essential for managing phase separation and the morphology of the composite nanoparticles. The thermodynamic incompatibility between the PVC and PS is responsible for the formation of PS bulges on the P(VC-co-AAEM) seeds. These anisotropic nanoparticles with non-cross-linked properties could potentially serve as compatibilizers for additional polymer processing. Wang et al. [45] studied the synthesis of graft copolymers using PVC as a toughening agent, and a mixture of isoprene, styrene, and methyl methacrylate monomers (MIS), with a core-shell structure through seed emulsion polymerization, evaluating the toughness, sub-micro-morphology, and dynamic mechanical behavior of the blends; they revealed that the impact strength of the blends was optimized when the content of MIS in PVC/MIS blends was 8 wt% and the content of isoprene in MIS was 70 wt%; the blends exhibited a typical ductile fracture due to the rubber particles’ toughening effect. The dynamic mechanical behavior of MIS-toughened PVC blends showed a more pronounced rubber peak than methyl methacrylate-butadiene-styrene (MBS), explaining the better toughening effect of MIS compared to MBS.

Mehrzad, Jamaati and Dorfeshan [46] studied the emulsion polyvinyl chloride (E-PVC) droplets in a spray dryer to understand the resulting dry powder’s morphology. E-PVC production involves turning VCM into liquid under pressure and a high temperature, then emulsifying it in water with the help of an emulsifier and polymerizing it using water-soluble catalysts. Parameters, including inlet air temperature, atomization ratio, and latex concentration, were studied to understand their impact on outlet temperature and powder moisture content, showing that more than 90% of E-PVC particles were solid spheres, and the remaining particles had various shapes. The particles’ final deformation was due to spray parameter changes during the drying process. Chen et al. [47] aimed to synthesize well-dispersed PVC/nanometer calcium carbonate (nano-CaCO_3_) composites. They used emulsifier tx10 polyoxyethylene octylphenol ether to modify nano-CaCO_3_ and create a uniform dispersion in water. A new method for characterizing the emulsion liquid was developed, which was used to synthesize PVC/nano-CaCO_3_ composites. The microstructure, particle size distribution, and thermal properties of the composite were analyzed using TEM, laser particle size analyzers, and thermogravimetry tests. The results showed that nano-CaCO_3_ was uniformly distributed in the PVC with a concentration of 5.2 wt%. Compared to unmodified PVC, the composite exhibited a more uniform particle size distribution and improved thermal stability. Gharieh, Mahdavian and Salehi-Mobarakeh [48] focused on improving the toughness of rigid polymers like PVC by designing impact modifiers that can be blended with the polymer matrix. Core-shell type impact modifier particles with different glass transition temperatures and nanometric shell thickness were prepared through seeded emulsion polymerization. The core comprised polybutadiene particles, while the shell was made of poly(methylmethacrylate-co-butyl acrylate) grafted onto the seed particles. The polymerization reaction was optimized, and the latex particles were characterized using various techniques. The core-shell particles had diameters of around 350–360 nm and glass transition temperatures ranging from 70 to 120 °C. The particles were blended with PVC, and the impact strengths were measured. The results showed that decreasing the shell’s glass transition temperature (T_g_) improved the impact resistance of the molded sheets, and the brittle-ductile transition temperatures increased with lower T_g_ values.

A variant of the emulsion process is the dispersion or microsuspension process. In this method, water, monomer (with a monomer-soluble catalyst), and surfactant (potentially combined with other agents) are passed through a high-pressure homogenizing pump before being introduced to the polymerization reactor. The kinetics of this process are mainly those of the suspension process because microsuspension is essentially a suspension process in which the droplets are incredibly small, resulting in unique properties for the produced PVC. Ji et al. [49] investigated the foaming properties of PVC plastisols derived from various PVC paste resins with different degrees of polymerization. These resins were produced using two methods: seed emulsion and micro-suspension. The study found that the seed emulsion method led to the best foaming quality for the formulation S5, while the micro-suspension method produced the best plastisol samples for the formulation S6. Low degrees of polymerization in seed emulsion-produced paste resins were more suitable for creating high-quality foam materials. The foam quality was judged based on the initial decomposition temperature and other parameters, with higher values indicating better foaming effects. The degree of polymerization and particle morphology influenced the rheological parameters of PVC plastisol and, consequently, the foam quality.

### 2.3. Bulk Polymerization

Bulk polymerization is a method of producing PVC in which the reaction takes place in the absence of solvents or dispersing agents [50]. The process can be divided into three main stages: initiation, propagation, and termination. During initiation, an initiator such as benzoyl peroxide is added to the VCM monomer to create free radicals that initiate the polymerization process. The free radicals react with VCM monomers to form PVC radicals, which then propagate and grow longer through the addition of more VCM molecules. Finally, the termination stage occurs when two PVC chains combine to form a PVC molecule, effectively stopping the polymerization process. According to Törnell and Uustalu [51], in agitated bulk polymerizations of VCM, primary particle formation occurs in two stages. The first stage takes place at the start of the process, while the second stage begins when initially formed particles start to agglomerate, continuing until at least 7% conversion is reached. Primary particles remain stable until they attain a certain size, which is smaller at increased stirring speeds. The number of particles created in the first stage does not depend on agitation or other polymerization factors. The second stage’s particle formation rate is equal to the particle agglomeration rate, which determines the overall number of primary particles formed. Initiation is critical for starting the reaction and controlling the rate of polymerization; in the propagation stage, the free radicals formed during initiation react with VCM monomers to form PVC radicals [52].

In the propagation stage, PVC radicals then continue to propagate and grow longer by the addition of more VCM molecules, forming a polymer chain. The rate of propagation is influenced by several factors, such as temperature, initiator concentration, and monomer concentration. The rate of propagation is also dependent on the chain transfer reactions that occur during the reaction. Chain transfer agents can be added to the reaction mixture to control the chain length and molecular weight distribution of the polymer; finally, the termination stage occurs when two PVC chains combine to form a PVC molecule, effectively halting the polymerization process. The termination reaction can occur through several mechanisms, including recombination of free radicals or disproportionation. In bulk polymerization, the termination reaction is typically governed by a combination of the chain transfer and combination reactions. The final PVC product can then be isolated through various techniques, such as filtration or centrifugation [53]. One of the main advantages of the bulk polymerization method is its simplicity and low cost, as it does not require any additional solvents or dispersing agents. However, the process can be limited by issues such as heat build-up, which can cause thermal degradation of the polymer and affect the quality of the final product. Moreover, controlling the molecular weight distribution of PVC can be challenging, as it depends on various factors such as the reaction temperature, initiator concentration, and monomer concentration [54].

Advances in the bulk polymerization are focused on the development of alternatives to free radical polymerization. Zhai et al. [55] crosslinked PVC foams with quantitative isocyanurate from the toluene diisocyanate trimer (tTDI) which were synthesized through bulk polymerization of toluene diisocyanate (TDI) in the presence of potassium stearate. The chemical structures and the final cross-linking network of the foams were characterized using FT-IR and NMR. The results showed that when fresh tTDI was used, no additional tTDI was detected, confirming the integration of the quantitative isocyanurate structure in the PVC foams’ crosslinking network. The T_g_ of the foams was determined by DMA. In the absence of phthalic anhydride, an inflection point appeared at about 10% in the tTDI content vs. the Tg curve, while the T_g_ of foams with phthalic anhydride increased linearly with the trimer content. Tsuchiya, Nomaguchi and Endo [10] investigated the bulk polymerization of PVC using the CpTi(OPh)3/MAO catalyst, producing high molecular weight PVC with good yields. The molecular weight of the polymer (M_w_) increased in direct proportion to the yield, while the M_w_/M_n_ ratio decreased with the increasing polymer yield. The PVC structure obtained was regular and its thermal stability was higher than that of PVC obtained from radical polymerization. The initial decomposition temperature of PVC produced with the CpTi(OPh)_3_/MAO catalyst depends on the polymer’s molecular weight, unlike PVC obtained from radical polymerization. We performed a cobalt-mediated radical polymerization (CMRP) of VCM using bis(acetylacetonato)cobalt(II) (Co(acac)_2_) as a controlling agent. An alkyl-Co(III) compound was employed as an initiator for bulk polymerization under non-isothermal conditions. The resulting PVC’s 1H NMR spectra revealed that the CMRP process did not significantly impact the defect levels compared to conventional free radical polymerization at the same temperature. Additionally, the copolymerization of VCM and VAc was controlled at 40 °C using the same alkyl-cobalt(III) compound, provided that sufficient VAc (about 40 mol%) was present in the polymerization medium to moderate the VC polymerization. Reactivity ratios showed that VC was preferentially incorporated in the polymer during the early stages of polymerization, resulting in copolymers with high VC content at moderate conversions.

### 2.4. Comparison of Polymerization Methods

Suspension polymerization, emulsion polymerization, and intrinsic (bulk) polymerization are distinct methods for producing polymers. Table 1 shows a comparative summary of these methods.

Each method has unique advantages and limitations, making their selection dependent on the specific polymer and application requirements. Considering the above, the preferred polymerization method for PVC production is suspension polymerization, because vinyl chloride is hydrophobic and not water-soluble and during the stirring the monomer is as droplets in an aqueous phase. In addition, the s-polymerization results in high-purity PVC, allowing a better particle size control, with a lower environmental impact compared to some other methods and is highly economical due to the possibility of recycling the unconverted MVC as raw material [68]. The end result of suspension polymerization is PVC in the form of a white powder, or resin, which is non-toxic, odorless, and inert.

## 3. Materials and Methods

This section describes the procedure for developing the bibliometric and co-occurrence study of process system engineering (PSE) applied to the production process of polyvinyl chloride (PVC), using VOSviewer software version 1.6.19 based on data obtained from the SCOPUS platform.

### 3.1. Bibliometric Analysis

**Figure 1:**
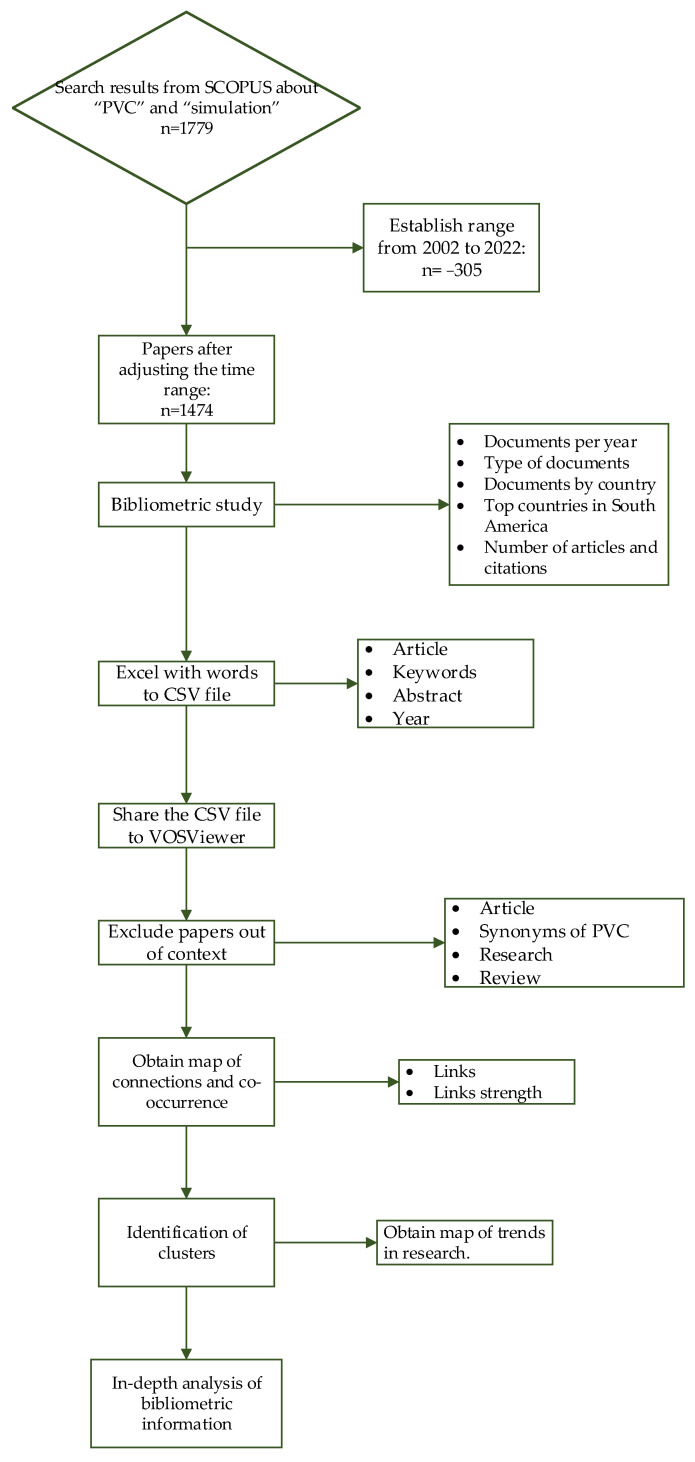
Flowchart describing the paper collection and bibliometric analysis process.

For this study, an initial data collection was conducted in the SCOPUS database related to PVC production and process systems engineering (Figure 1).

The purpose of this search was to contextualize the implementation of PSE for design, evaluation, control, and optimization of the production process, involving an exploratory search for existing research on the relationship between PVC and process simulation. A systematic search was carried out in SCOPUS, focusing on the terms within the title, abstract, and keywords of the articles using the terms “PVC” and “Simulation”. The term PSE was avoided, given that it can have different meanings such as “Public Service Enterprise”, “Pseudoephedrine”, “Power Systems Engineering”, “Personal and Social Education”, among others, which introduces noise to the word map and affects the analysis. For the word PVC, some out-of-context definitions were discarded manually, such as “Premature Ventricular Contraction”, “Permanent Virtual Circuit”, or “Precessing Vortex Core”. The selected time interval was from 2002 to 2022, taking into account that 2023 is ongoing.

With the information obtained in SCOPUS, the number of research documents, including research papers, conference proceedings, books, book chapters, and review papers per year, was obtained and analyzed. Following this, the distribution of documents published by type was obtained to identify the priority of original research and findings in the publication trends, as well as the role of conferences and professional gatherings in spreading knowledge related to PSE applied to PVC production. Subsequently, the 15 highest document-producing countries and the number of publications of each country from 2002 to 2022 were obtained and analyzed, comparing the results with top PVC-producing countries, and examining the trends by continent. A more detailed analysis was made for the case of South America, showing the top five document-producing countries to observe regional impacts and tendencies. In addition, the number of research and review papers per year in contrast to the number of citations for the past 15 years was obtained and compared to identify correlations in the variation of both indicators.

### 3.2. Co-Occurrence Study

Using the same information obtained in the bibliometric analysis, keyword co-occurrence was evaluated with the VOSviewer software version 1.6.18 [69].The minimum number of keyword co-occurrences handled was set at five. Subsequently, frequent keywords unrelated to the scope of this paper were identified and eliminated through a careful filtration process. After we had completed the filtration, a map of connections and co-occurrences was generated, showcasing the frequency of keyword appearances, the connections between keywords with an emphasis on the number of links and the link strength between two keywords, and the clusters of words. These word clusters were grouped by colors to facilitate the identification of connected research trends. The most relevant clusters were then selected for further in-depth analysis.

Another word map was obtained to illustrate the variation in research trends over time in relation to PSE applied to PVC production processes. For this study, the time interval was set at 10 years, taking into consideration limitations in the software. The analysis involved identifying connections between keywords for specific periods of time, observing the evolution of the research landscape, examining changes in priorities within the scientific community related to research in PSE and PVC, and tracking ongoing advancements in these fields.

### 3.3. In-Depth Analysis

Once the co-occurrence analysis was completed and the word clusters prioritized, an in-depth analysis was conducted to gain a deeper understanding of the most relevant and groundbreaking works developed in the field. This analysis involved reviewing each document within the selected clusters, summarizing the content, and highlighting key aspects, such as findings, methods, conclusions, and future work presented by the authors. In this stage, relevant documents from the ongoing year (2023) were also incorporated to ensure that the analysis captured the most recent developments in the field. The results obtained from this comprehensive analysis were then clustered into groups based on the aim and scope of the studies, taking into account the contributions made in various aspects related to the PVC production such as reactor and process modeling, design and evaluation of PVC production processes using PSE, process control and optimization, and evaluation of the process under safety and sustainability parameters.

## 4. Results and Discussion

Next, the bibliometric and co-occurrence analysis of the process system engineering (PSE) applied to the production process of polyvinyl chloride (PVC) was analyzed.

### 4.1. Bibliometric Analysis of “Simulation” and “PVC”

**Figure 2:**
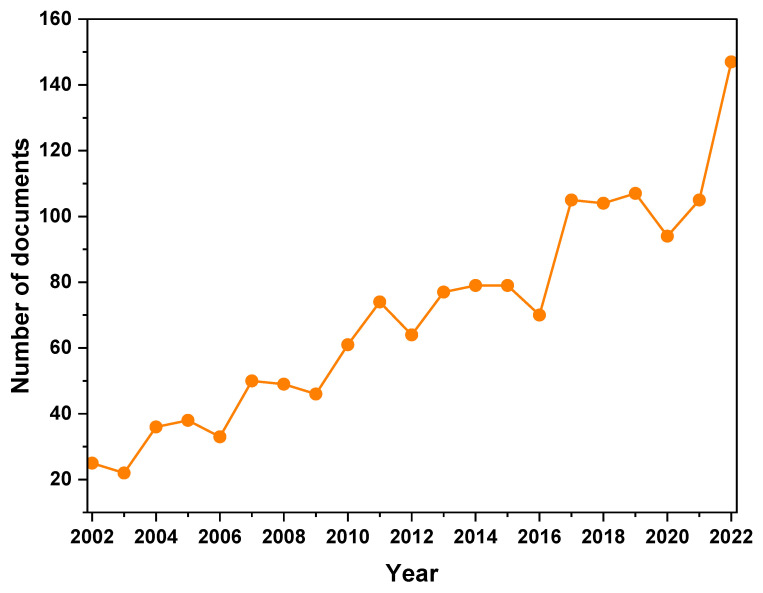
Number of documents published per year regarding simulation and PVC.

After the search made in SCOPUS with the search equation selected, 1662 documents were found; once we had refined the search by excluding from the keywords in VOSviewer documents related to medicine, the total number of documents decreased to 1458. There has been a general increase in the number of publications over the years, indicating a growing interest in research related to “simulation” and “PVC”; the most significant jump in the number of publications occurred between 2016 and 2017, from 70 to 105, representing a 50% increase in the number of documents; it can also be observed that the year 2020 had a relatively lower number of publications compared to the surrounding years, with 94 documents; it can be argued that this decrease may have been a result of the public health emergency that occurred that year. However, the highest number of publications was in 2022, with 147 documents, demonstrating a continued growth in research interest and activity. The period from 2002 to 2006 had relatively fewer publications compared to later years, suggesting that research interest in the subject was still emerging during that time (Figure 2).

The results shown in Figure 2 evidence a fluctuation in the number of publications about PVC and simulation over the years due to a variety of factors. This could be due to many reasons, such as the availability of funding that can greatly influence the number of research publications. Increased funding in a particular year can lead to more research and subsequently more publications. In addition, the development of new technologies or methodologies can spur interest in a particular field, leading to an increase in research activity and publications. Events such as the COVID-19 pandemic could have affected research activities and priorities, possibly leading to a decrease in publications in 2020. In addition, the rise in 2021–2022 could be due to renewed interest in the field, possibly driven by new applications or breakthroughs. According to the growth pattern of PVC-simulation-related research, the number of publications will continue to grow in the future.

**Figure 3:**
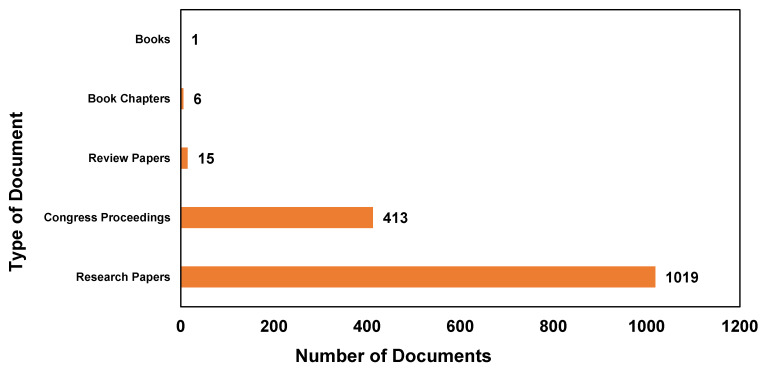
Distribution of document types indexed in Scopus with keywords “simulation” and “PVC” from 2002 to 2022.

Figure 3 illustrates the distribution of document types indexed in Scopus, featuring the keywords “simulation” and “PVC” over the past 20 years. A comprehensive analysis of the data demonstrates that research papers predominate, accounting for 1019 documents, thus emphasizing the significance of original research and findings in this domain. Furthermore, the data reveal that 413 congress proceedings contribute substantially to the overall publication count. This observation underscores the vital role that conferences and professional gatherings play in disseminating knowledge, fostering collaboration, and facilitating the exchange of ideas within the scientific community. In contrast, review papers, which primarily focus on summarizing and synthesizing existing research, account for a comparatively smaller portion of the publications, with a total of 15 documents. This indicates that, although there is interest in collating and examining the current state of research in this field, it is not as prevalent as original research and conference proceedings. Lastly, a minimal number of book chapters (six) and books (one) has been published on this topic, indicating that research in this domain is predominantly disseminated through research papers and conference proceedings, as opposed to books or book chapters.

“Research papers” and “congress proceedings” often constitute the largest quantity of published documents in a given field due to their depth and rigor because research papers typically contain completed research that has undergone extensive review by experts in the field through a blind reviewing process. This makes them a reliable source of detailed and rigorous information, leading to a high volume of such publications. Furthermore, the growth in science is driven by the publication of novel ideas and experiments, most usually in peer-reviewed journals. This leads to an increase in the number of published papers over time.

In addition, conferences are a vital platform for researchers to present their work, obtain feedback, and network with other professionals in their field. The proceedings of these conferences, which include the presented papers, are often published to disseminate the research discussed at the conference; hence, conference proceedings can often be published more quickly than journal articles, allowing for timely dissemination of research findings. These factors contribute to the high quantity of “research papers” and “congress proceedings” about PVC and simulation from 2002 to 2022.

**Figure 4:**
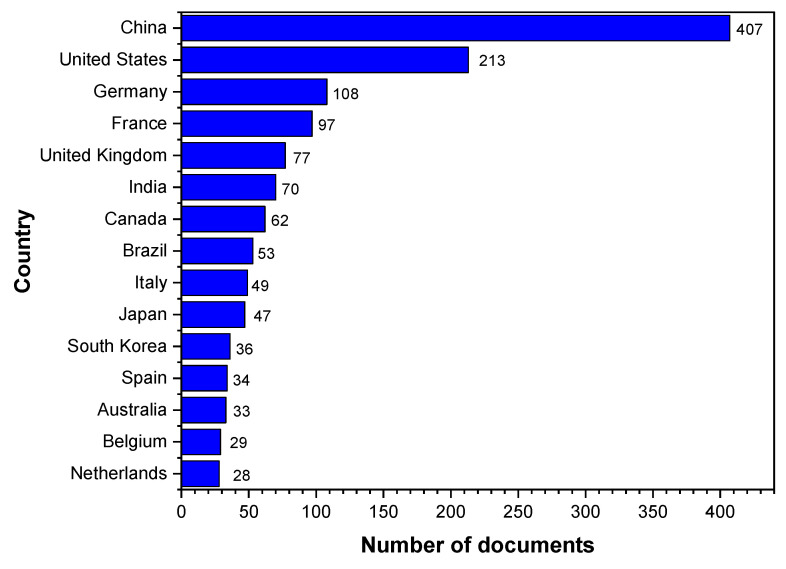
Number of publications by top 15 countries regarding simulation and PVC from 2002 to 2022.

Figure 4 shows the top 15 document-producer countries and the number of publications from 2002 to 2022. From the data presented in Figure 3, it can be observed that China leads the research output with 407 documents, followed by the United States (213), Germany (108), France (97), and the United Kingdom (77). This may be attributed to several factors, including the size of the economy, investments in research and development, and the presence of a strong polymer and plastics industry. According to Statista [70], the largest PVC-producing countries are China, the United States, Germany, Japan, South Korea, India, Brazil, France, the United Kingdom, and Canada. Comparing the research output in Figure 3 with the highest PVC-producing countries, a general correlation between the two rankings can be observed. The top three PVC-producing countries, China, the United States and Germany, are also the leading countries in terms of research output in the field of “simulation” and “PVC”; this correlation suggests that countries with a strong PVC industry tend to invest more in research and development, which may result in higher numbers of publications. These countries may also have well-established research institutes and infrastructure, enabling advancements in simulation techniques for PVC applications. Developing countries such as India and Brazil also have a notable number of publications (70 and 53, respectively), suggesting an emerging interest in this field, which could be driven by an increasing demand for PVC products and a need for advanced simulation techniques in their industries.

Analyzing Figure 4 by continent, it can be observed that Asia leads the research output with 560 documents, followed by Europe (426) and North America (275). Asia’s dominance in research output can be attributed to the contributions of China (407), Japan (47), South Korea (36), and India (70). China has a significant impact on the overall research output in Asia. This can be linked to its large population, economic growth, and investment in the PVC industry. Europe’s research output is driven by contributions from Germany (108), France (97), the United Kingdom (77), Italy (49), Spain (34), Belgium (29), and The Netherlands (28). The European Union’s emphasis on collaborative research projects and the presence of established research institutes likely contribute to this trend. North America’s research output is primarily due to the United States (213) and Canada (62). Both countries have well-developed research infrastructure, strong economies, and a significant polymer and plastics industry. Australia/Oceania and South America have comparatively lower research outputs, with 33 and 53 documents, respectively. This may be due to smaller economies, lesser investments in research and development, or different priorities in their industrial sectors.

**Figure 5:**
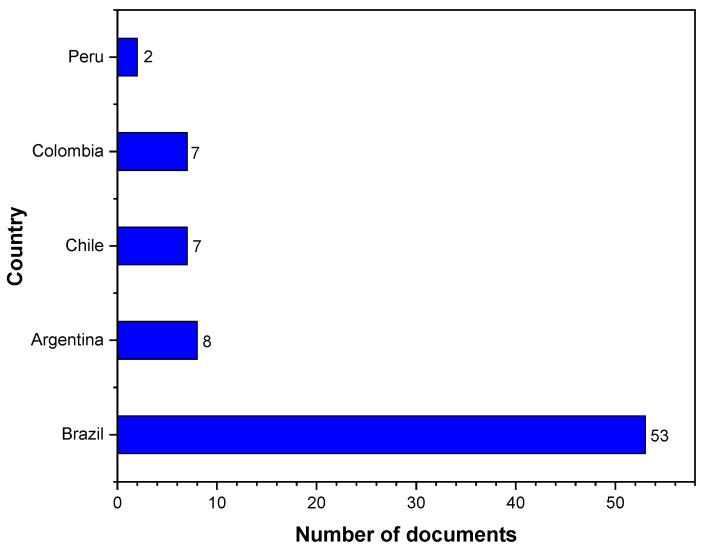
Number of publications by top five countries in South America regarding simulation and PVC from 2002 to 2022.

Figure 5 shows the top five countries in South America with contributions via research documents related to PVC and simulation for the period 2002–2022. In South America, the country with the most publications on the subject is Brazil with 53 articles; Argentina has 8 publications and Colombia has 7 publications, as does Chile. It can be concluded that although Colombia is in the top five and very close to the top two, more research efforts related to the topic are needed to approach Brazil’s level.

The high number of documents published about PVC-simulation in Brazil and Argentina could be attributed to government policies; hence, both countries may have policies that encourage research and development in the field of PVC and simulation. This can lead to an increase in the number of published documents. In addition, Brazil and Argentina have significant industries related to PVC production and use, which could stimulate research and result in a higher number of publications. Another reason is the presence of strong academic and research institutions in these countries that can also contribute to a high volume of research output. PVC is known to have environmental and health impacts [71]. The high number of publications could be a response to these concerns, with researchers seeking ways to mitigate these impacts through simulation and other methods.

**Figure 6:**
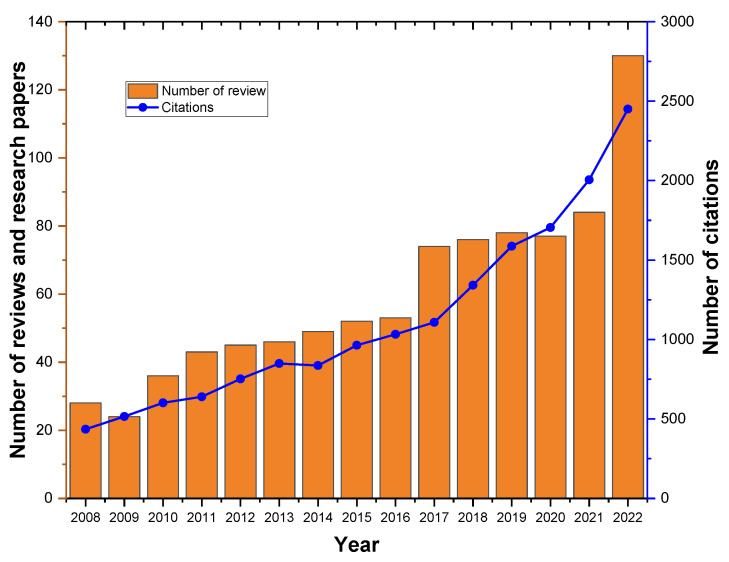
Number of research and review papers and citations of these documents from 2008 to 2022.

In Figure 6, we can see the number of both research and review papers by year in contrast to the number of citations of these kinds of documents from 2008 to 2022; this figure is made for the past 15 years by limitations in the information given by SCOPUS. From this combined graph, it can be analyzed that in general terms, as the number of published articles on the subject has increased, so has the number of citations. However, in 2014, the number of citations was slightly lower than in 2013. For the years 2020 and 2021, the citation growth rate was higher than the article publication growth rate. This can be explained by to the health emergency during these two years, which often prevented access to both experimental and computational laboratories for research. However, access to article databases remained open remotely in many places, allowing for the citation of already published articles. In 2022, with the resumption of activities, articles in progress were unstuck, and there was an increase of almost 25% in the number of publications compared to the previous year, surpassing the citation growth rate for this year.

Based on the output and citation counts, PVC-related simulation research is generally favored by large-scale journals. However, the sizes of the research effect are field-specific. The improvement in and sophistication of the fields of computer science and engineering have paved the way for the development of this topic. These results are consistent with the findings of the top keywords of the included paper.

### 4.2. Co-Occurrence and Research TRENDS Study

**Figure 7:**
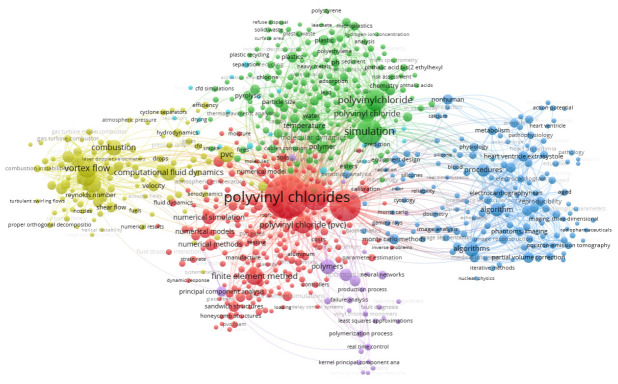
Map of connections and co-occurrences between keywords for simulation and PVC.

From the keywords extracted from SCOPUS to the VOS viewer for generating the map of connections and co-occurrences (Figure 7), the frequency of appearance of the keywords, the connections between keywords, and the clusters of words were analyzed. It can be seen that the most repeated ones, excluding PVC or simulation and their synonyms, are “Chlorine compounds”; this allows us to infer that PVC is studied based on its chemical nature, grouped into a series of compounds containing chlorine, an element to be studied for its toxicity as a gas and its abundance when combined with other elements such as salts or organochlorine compounds. Another frequent word and the first closely related to the trend we want to find is “algorithm” which shows that systematic operations, either quantitative or qualitative, are being developed and used to solve problems related to PVC production. This can have many work areas, such as process control, product property prediction, reaction kinetics, models for recycling, or sustainability quantification. Another high-frequency term of interest is “computational fluid dynamics”, showing that computational fluid dynamics studies involving PVC are also a specific topic of interest. However, there may be a bias since if the studies are based on pipe geometry, PVC may simply be the material of the pipe.

Regarding connections between keywords, the analysis was made over words related to modelling, simulation, and algorithms. It can be noted that the word “simulation” is one of the most interesting and most frequent in this analysis, having the most links (526) and the highest link strength (2265). It is related to terms such as computational fluid dynamics, which show a line of work in simulation at this scale. It is also linked to terms like velocity, temperature, particle size, flow, etc., showing the use of simulation for evaluating or predicting operational conditions or process/production parameters. The numerical simulation item is linked to the words “finite elements”, which allows us to see the techniques used for this type of simulation. The word “optimization” is related to real-time control, showing the specific application given to this theme in the context of PVC production.

With respect to groups of words or clusters, five word groups could be identified in the map represented by the software with different colors, allowing us to confirm the trends to be deepened or discarded in further sections of this work. Cluster 1, in red, can be identified as related to mathematics and statistics, referring to analysis techniques such as failure analysis, artificial neural networks, least squares approximation, fuzzy logic, etc. Cluster 2, in blue, is related to the biomedical field, which may be PVC applications or algorithms for medical result analysis because it contains key terms such as anatomical model, biomechanics, blood, computer-assisted diagnosis, computer-assisted tomography, etc. which are outside the scope of this work. Cluster 3, in green, is related to polymer chemistry and some separation phenomena, including terms such as activation energy, adsorption, chemical analysis, chemical industry, degradation, diffusion, kinetics, among others. Cluster 4 is related to polymer applications with terms such as pipes, bottles, foams, reinforced concrete, mechanical properties, and, from the simulation aspect, computational simulation, numerical methods, and Monte Carlo simulation, among others. This cluster contains words that makes noise in the analysis given by other uses of the word PVC such as “vortex flow”. Finally, cluster 5, in lilac, shows words repeated in other clusters or synonyms; for this reason, this cluster was discarded for in-depth analysis. From this evaluation, it can be said that areas of research of our interests are mathematics and statistics, with their applications to polymer chemistry, separation phenomena, and polymer applications.

**Figure 8:**
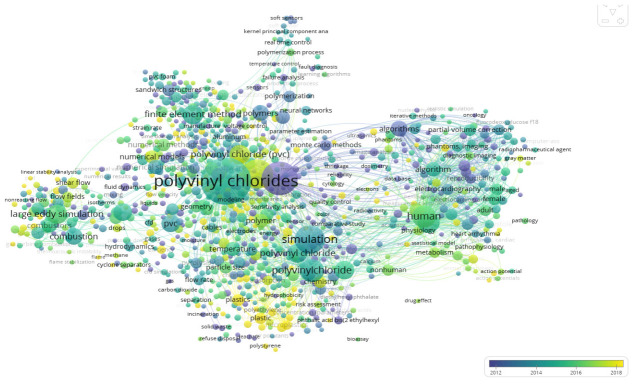
Map of trends in research with time for simulation and PVC.

For analysis of research trends with time (Figure 8) in relation to the search equation used for the analysis, it is evident that the simulation work associated with PVC experienced a surge in interest at the beginning of the last decade. At that time, the primary focus was on Monte Carlo methods and numerical simulation, which helped advance the understanding of PVC’s properties and behavior. Following this initial period, research in the field shifted towards biomedicine, with a growing interest in the analysis of results related to this domain. This transition corresponds to Cluster 2 in the previously discussed word cluster analysis. The shift indicates that researchers began to explore the potential applications of PVC in biomedical settings, as well as studying the material’s interactions with biological systems. More recently, research on PVC and simulation has expanded into various areas, including the broader study of plastics and microplastics. This trend highlights a growing concern for the environmental impact of plastic materials and the need for sustainable solutions. Additionally, the development and application of new algorithms have become increasingly important, as researchers seek to improve the accuracy and efficiency of simulation methods across diverse applications. As a result, the research landscape has evolved significantly over time, reflecting both the changing priorities of the scientific community and the ongoing advancements in the field.

### 4.3. In-Depth Analysis of the Bibliometric Information

According to results of co-occurrence and a research trends study, the in-depth analysis of the research was focused on the modelling and simulation of PVC production processes, including the development of models for understanding the dynamic behavior of the different process units and the overall production system as well as the use of simulation tools in the design and evaluation of PVC production processes, the process control and optimization in improving the performance of PVC production, as well as the development of analysis and optimization strategies that take into account multiple objectives such as product quality, energy efficiency, safety, and sustainability.

#### 4.3.1. Modeling and Simulation of PVC Production

The primary focus of these studies has been on the modeling of the polymerization reaction, as the operating conditions of this stage have a critical influence on the characteristics and properties of the polymer. Various aspects, such as reaction kinetics, thermodynamics, polymer properties and characteristics, process variable control, and operating times, have been extensively investigated to gain a deeper understanding of the PVC polymerization process. In the study by Xing et al. [72], a neural network soft-sensor model with data dimensionality reduction strategies was proposed for predicting the conversion rate of VCM in the PVC production process. To address the issues of complex neural network topology and lengthy training time, seven data dimensionality reduction methods were employed. These methods helped reduce the high-dimensional input data used in the neural network soft-sensor model. The radial basis function (RBF) neural network and the dynamic fuzzy neural network (D-FNN) were then utilized to predict the VCM conversion rate. Simulation results demonstrated that the proposed neural network soft-sensor models effectively predicted key economic and technical indicators of the PVC polymerization process, meeting real-time control requirements for PVC production. Bárkányi et al. [73] explored the size distribution of monomer droplets in a batch reactor by utilizing a population balance model and the Monte Carlo method implemented in MATLAB software. Their research demonstrated that the characteristics of the monomer droplets within the reactor play a crucial role in polymer formation. They also found that the agitation process directly impacts the coalescence of the droplets, ultimately affecting the particle size distribution.

Kiparissides et al. [74] made further advancements in process modeling by developing an integrated multiscale and multiphase model. This model aimed to describe the dynamic operation of PVC suspension polymerization and the properties of the generated polymer in a batch reactor. By considering the reaction kinetics, thermodynamics, and transport phenomena (mass and energy) of the suspension-reactor system, they provided a detailed mathematical formulation of macroscopic and microscopic phenomena. Moreover, a population balance model was incorporated to analyze the evolution of particle size throughout the operation. They also showed that their model could adequately describe the reactor operation and accurately represent properties such as molecular weights and characteristics like the size and porosity of the polymer during the reaction. Kiparissides and Pladis [75] critically examined the role of primary and secondary stabilizers in the suspension polymerization of VCM and their impact on grain morphology, specifically particle size distribution, grain porosity, and bulk density. The researchers employed the modified Mooney equation to calculate the time-varying viscosity of the suspension in an SPVC reactor based on the volume fraction of the dispersed monomer/polymer phase, the viscosity ratio of the dispersed phase to the continuous aqueous phase, and the maximum packing volume fraction of S-PVC grains. They also numerically solved a population balance equation to estimate the dynamic evolution of particle size distribution (PSD) in an industrial batch suspension VCM polymerization reactor. A porosity model was postulated to calculate the dynamic evolution of PVC grain porosity concerning monomer conversion and primary particle fusion extent. Additionally, the study detailed a theory for calculating the required agitation power in S-PVC reactors, showing that the time-varying viscosity of the suspension and the PVC grain morphology (extent of particle agglomeration) can be assessed through on-line estimation of the effective volume fraction of the dispersed phase using on-line power agitation measurements.

Moreira and Pires [76] developed a model and simulation of an oxychlorination reactor with a fluidized bed, focusing on 1,2-dichloroethane, which serves as an intermediate in the synthesis of VCM. Utilizing a pseudo-homogeneous model with one-dimensional flow in a steady state, the researchers based their approach on the two-phase fluidized bed theory. This enabled them to perform sensitivity analysis on various operational and design parameters of the reactor. The mathematical model’s system of ordinary differential equations was solved using the Newton–Raphson numerical method. Despite the employment of a one-dimensional model, the results showed that it appropriately represented the system. Through sensitivity analysis, the study identified the most influential parameters affecting reactor performance, including fluidized bed height, bubble diameter, residence time, cupric chloride weight in the catalyst, and emulsion phase temperature. Pakdel et al. [77] used experimental and mathematical methods to investigate the emulsion polymerization of VCM, developing a computer code based on the zero-one population balance to analyze the effects of the initiator and emulsifier concentration on VCM conversion during polymerization. The model was also designed to capture particle coagulation, enabling a reliable evaluation of the PSD. The rates of homogeneous and micellar nucleation mechanisms were simulated, predicting changes in the PSD and the number of polymer particles influenced by the initiator and surfactant concentration. The model results were consistent with experimental findings and clearly demonstrated the impact of these concentrations on the PSD of prepared PVC latexes.

Kiparissides et al. [78] introduced novel software that enables the modeling of dynamic behavior in suspension polymerization reactions within a batch reactor. This advanced mathematical approach considers various factors, including reaction kinetics, thermodynamics, and reactor parameters, such as geometry and controllability. For validation of the software, the researchers carried out laboratory tests and compared the outcomes with the software-generated results. The analysis revealed a good fit between the model and the experimental data, indicating that the software can accurately describe the polymerization reaction, encompassing aspects like conversion and concentration. It can also predict the characteristics of the produced polymer, including average molecular weights. This functionality is significant, as it aids in understanding and optimizing the polymerization process for desired polymer properties. Ibrahim et al. [50] proposed a new dynamic mathematical model for the three-phase structure model, in a bulk polymerization of PVC, considering all particles in the gel, solid, and liquid phases, with an emphasis on using mercury as a catalyst. The model considers the heat and mass transfer between phases and chemical reactions that occur within them. The effects of the catalyst and volumetric flow rates of the VCM were evaluated using the proposed model, and findings regarding concentration and temperature reaction were compared with experimental data, showing good agreement between the proposed mathematical model and plant data.

Lewin [79] delved into the effects of cooling and the addition of initiators on the operation and quality of polymers during suspension polymerization reactions in a batch reactor. By employing mathematical models adjusted with data from industrial plant operations, it was found that the initiators’ addition impacts the suspension’s temperature. Therefore, this addition should be correlated with the cooling liquid’s temperature. Additionally, the study determined that the quantity of initiators plays a critical role in temperature variation and reaction time. Mejdell et al. [80] applied a rigorous model to examine the cooling system of a PVC reactor during industrial-scale polymerization, utilizing the method of characteristics. The model aimed to predict conversion and reaction kinetics through heat balance. Although successful in predicting the reactor’s thermal conditions, the model displayed irregularities in accurately predicting reactor kinetics, indicating a need for more detailed mathematical developments concerning kinetics. Hao et al. [81] focused on the suspension polymerization process of PVC in a 50 m^3^ stirred tank reactor. They used conventional design methods for a structural design and established a standard K-E turbulent calculation model based on fluid dynamics theory. The study employed FLOTRAN CFD software to simulate complex flow fields and flow patterns, providing insights into optimizing a reactor design. In the study of Shurrab et al. [26], data envelopment analysis (DEA) and the theory of constraints (TOC) were combined to address bottlenecks in a PVC pipes production line. Four scenarios were tested to alleviate the bottleneck constraint, with DEA being used to identify the optimal solution. The best solution involved upgrading the constraint, which resulted in significant performance improvements. Although this solution increased costs by 20%, it boosted the annual PVC pipe production by 21%, raised line throughput from 0.3 pipe/min to 1.15 pipe/min, and improved system utilization by 19%. Wieme et al. [82] created a model for the comprehensive examination of the operating conditions (kinetics, thermodynamics, heat transfer, and PID control) of a suspension polymerization reactor at laboratory and industrial scales. The model was adjusted to fit the data reported in the literature. It was discovered that proper temperature control is crucial for the reaction to progress adequately, particularly at the industrial scale. In a study by Gao et al. [83], the authors tackled the issue of pressure fluctuations within the nose during extrusion molding, which is a significant problem in extruder production. Using CFX fluid simulation, they investigated the flow field distribution in extruders based on PVC production. The researchers were able to determine the relationship between screw speed and extrusion pressure, providing valuable guidance for controlling pressure inside the extruder.

Rodrigues et al. [84] modelled a large industrial PVC batch reactor across various operating conditions, including temperatures, grades, and initiators, covering the entire monomer conversion range. A first-principles model, primarily based on a priori equations and parameter values, was developed. The model relies on Xie and Hamielec’s kinetic model for PVC production by suspension polymerization, accounting for gel, glass, and cage effects on termination, propagation, and initiation kinetic constants [85]. Some kinetic model parameters were adjusted to match plant data. The simulation model includes energy balances for the reactor system and a temperature feedback control scheme. Simulation tests were run covering multiple polymerization temperatures and initiators, encompassing all operating ranges of interest for industrial conditions.

Gao et al. [86] focused on developing a real-time fault diagnosis and optimized monitoring strategy for polymerizers in the PVC production process, combining rough set (RS) theory, an enhanced discernibility matrix, and a back propagation (BP) neural network. By employing the improved discernibility matrix, they effectively minimized the input dimensionality of fault characteristics through rough set attribute reduction. The fuzzy C-means clustering algorithm was used to discretize the continuous variables in the decision table. The Levenberg–Marquardt BP neural network was then trained based on the simplified decision table to establish the configuration parameters for the proposed polymerizer fault diagnosis model, facilitating the nonlinear mapping from fault symptom sets to polymerizer fault sets. The effectiveness of the proposed rough set and BP neural network-based fault diagnosis approach was demonstrated through simulation experiments using industry historical data.

In the literature, various studies have simulated PVC production processes, focusing on stages beyond the reactor (downstream). For instance, Hoa et al. [87] conducted a simulation using SuperPro Designer software for the stage of removing unconverted VCM from the suspension in a gasifier tank and columns. This process successfully eliminated 99% of the monomer from the mixture. Additionally, the drying stage was simulated with a decanter tank and a spray dryer, which removed 99% of the water from the PVC. This study was designed for a production plant with a capacity of 118 tons per day, featuring 10 reactors processing 7.7 tons of MVC at a 90% conversion rate. Beltrán Domínguez [88] designed and simulated a PVC production plant capable of producing 56,000 annual tons with an 85% conversion rate. The process included MVC removal and drying stages, but residual MVC was not treated. This study also involved calculations to determine the cost of the equipment. Mangili et al. [89] carried out a simulation of the MVC production process using Unisim software, along with an assessment of the water consumption index in the process. The quantification of the index was based on the losses of water streams in the utility systems of the process (liquid and steam), including heating, cooling, and other related systems. It was determined that the cooling process had the highest liquid consumption, with 2696 m^3^/h and a 3% loss percentage (80.90 m^3^/h). In contrast, the water used as steam had a lower consumption rate (67 m^3^/h) but presented a higher 10% loss. This indicator had a value of 0.88 m^3^ per ton of MVC, suggesting the need for adjustments to the system through process integration.

Astorayme et al. [90] conducted a pre-feasibility assessment for a MVC and PVC production plant by suspension, using computer-assisted process engineering. The process was simulated in Unisim, with a production capacity of 200,000 annual tons of PVC. In addition, an economic analysis and a preliminary environmental study were carried out, finding a profitability of 81 million dollars per year and a payback period of 3 years. The toxicity of substances was also identified as a potential environmental impact. Karasek et al. [91] conducted a study in which they compared two MVC production processes: one by direct chlorination of ethylene and another through the oxychlorination of ethylene with hydrogen chloride (HCl). Both processes were simulated using Aspen Plus software, aiming to produce 5 tons of MVC per hour. In the case of the direct chlorination process, a production yield of 95.4% was obtained; however, it generated an HCl byproduct stream (3250 tons per hour). On the other hand, the oxychlorination process achieved a 97% efficiency and did not present byproducts, as the residual HCl was recirculated in the process. In addition to the simulation of the processes, pinch analyses, environmental assessment using the Biwer method and a pre-feasibility economic analysis were carried out. These analyses revealed that the oxychlorination process presented better energy performance compared to the direct chlorination process. The oxychlorination process showed a lower energy requirement, with 5 × 10^6^ kJ for heating and 1.54 × 10^7^ kJ in cooling, while the direct chlorination process required 1.6 × 10^7^ kJ for heating and 2.5 × 10^7^ kJ for cooling. From an environmental perspective, it was observed that the oxychlorination process had better performance due to the absence of hazardous byproducts, unlike the direct chlorination process, which generated an HCl byproduct stream. Finally, the economic analysis showed that the oxychlorination process had a higher economic appeal, with a payback period of 0.37 years and a net present value of USD 42.6 million over a period of 15 years. In contrast, the direct chlorination method presented a payback period of 0.77 years and a net present value of USD 14.4 million in the same period.

Gao et al. [86] focused on the challenges presented by the PVC polymerization production process, such as its nonlinear nature, multiple variables, strong coupling, and extensive time delays. To fulfill the real-time fault diagnosis and optimized monitoring needs for critical large-scale polymerization equipment, the authors developed a strategy that incorporated rough sets theory with an enhanced discernibility matrix and BP neural networks. By using the improved discernibility matrix, they effectively reduced the input dimensionality of fault features through attribute reduction. The Levenberg–Marquardt BP neural network was subsequently trained to identify polymerization faults based on the simplified decision table, facilitating the nonlinear mapping from fault symptom sets to polymerization fault sets. When combined with industry historical data in simulation experiments, the proposed rough set neural networks fault diagnosis approach proved effective, considerably increasing the accuracy and efficiency of the polymerization fault diagnosis system.

#### 4.3.2. Process Control and Optimization

Su and Wang [92] presented a process optimization approach for PVC production using the calcium carbide method in a hybrid system. The proposed integration model addresses uncertainties in scheduling by incorporating discrete-time methods and chance-constrained programming for planning. Continuous-time modeling and optimization of equipment-level operations with variable production rates are used for scheduling. The data-driven robust optimization method is applied to handle endogenous uncertainty. This approach improves the robustness and stability of the production system while reducing total costs and energy consumption. Miller et al. [93] modeled the temperature control system of a PVC suspension reactor using a nonlinear triple cascade system. This approach enabled the study to maintain the appropriate reaction temperature. Zhang et al. [94] developed a mathematical method to explore the polymerization process in industrial suspension polymerization reactors of VCM, taking into account both the polymer and reactor’s properties and conditions. The model’s results aligned with the experimental tests reported in the literature. De Paula and Martínez [95] proposed a novel simulation-based approach for dynamic optimization under uncertainty in discretely controlled continuous processes (DCCPs) using multi-modal Gaussian process dynamic programming (mGPDP). A key advantage of this approach is that it employs probabilistic models (Gaussian processes) to concurrently learn transition dynamics that describe mode execution and represent the optimal control policy for mode switching, instead of relying on an inefficient global metamodel. The researchers used throughput maximization and smoothness in a typical PVC production line, facing significant schedule variability due to resource sharing.

Kharlampenkov et al. [96] evaluated new aspects of technical equipment in PVC production, aiming to reduce the environmental impact of the process. Innovative technologies, such as plasma-chemical pyrolysis of coke or methane contained in coke gas, can help decrease energy and capital intensity. The development of cooperative ties between the coke chemical enterprise and chemical enterprises in the Kemerovo region was suggested to promote the clustering of the regional economy. The article also provides data on the cost of technological equipment, unit costs per unit of production, and the project cost o creating PVC production based on the raw materials of KOKS PJSC. Implementing the proposed technology can reduce the ecological burden on the region. Li et al. [97] analyzed the impact of VCM purity on PVC resin quality, highlighting the limitations of traditional VCM distillation systems. The authors proposed an optimized design involving changes in the refluxing mode, increased operating pressure for low and high boiling towers, the addition of high-boiling residue recycling equipment, and the replacement of tower internals. Through simulation and optimization, the purity of VCM improved from 99.95% to 99.999%, with significant reductions in both low- and high-boiling residues. The proposed modifications are expected to yield considerable cost savings and financial benefits, estimated at CNY 34.71 million per year for the tower transformation and CNY 5.83 million per year for the high-boiling residue recycling system. In the study of Hu et al. [98], a pilot-scale reverse osmosis (RO) system was developed alongside a membrane bioreactor (MBR) to desalinate and reuse wastewater from a PVC production site. The solution–diffusion–film model (SDFM) was proposed to describe the rejection of electrolyte mixtures in the MBR effluent, which contained dominant ions and trace ions. The universal global optimization method was used to estimate ion permeability coefficients and mass transfer coefficients in SDFM, and the membrane performance was evaluated based on these parameters. An energy analysis model was also proposed to analyze the specific energy consumption of the pilot-scale RO system in various scenarios, taking into account thermodynamic restrictions. The theoretical simulations were found to align with experimental results for dominant ions.

Su, Wang and Gao [99] developed a scheduling model for PVC production using the calcium carbide method, based on a continuous-time modeling approach. An improved mixed-integer nonlinear programming (MINLP) model is proposed, with the goal of minimizing total cost. To balance solution speed and quality, a combined algorithm using both the MINLP and approximated mixed-integer linear program (MILP) models is developed for PVC production scheduling. The optimization results from the linear model are used as initial values in the original MINLP model to speed up the solution process. The method’s effectiveness is verified using two real-world cases, showing significant improvements in computation speed and solution accuracy. Qi et al. [100] examined the challenges of controlling the PVC polymerization temperature in a chemical plant, given its complex characteristics and control difficulties. They proposed a new PID control method based on internal model control, which offers improved control against time delay and disturbance. By combining these two approaches, the authors aimed to achieve a more accurate and stable polymerization temperature control. Simulation results demonstrated the effectiveness of the proposed method, showcasing its enhanced stability, robustness, and ability to counteract disturbances. Wen et al. [101] developed a fault diagnosis strategy for polymerization kettles; a key device in the PVC production process is proposed using a support vector machine (SVM) and cuckoo search (CS) algorithm. By analyzing the PVC polymerization process, a mapping between process data and fault modes is established. The CS algorithm is then used to optimize the SVM’s penalty factor and kernel function parameters. This enables the nonlinear mapping from symptom set to fault set for fault pattern classification. Simulation experiments using industrial on-site historical data show that the CS-SVM fault diagnosis strategy is effective.

#### 4.3.3. Safety and Sustainability of the PVC Production Process

Ren et al. [102] addressed the importance of considering carbon pricing in PVC production, developing a stochastic mixed-integer linear programming model for PVC pipe manufacturing in China and assessing the impact of life cycle emissions and carbon market uncertainty on supply chain decisions. The study demonstrates that carbon market uncertainty significantly influences both carbon-intensive and downstream manufacturing sectors, leading to different procurement choices, supply chain configurations, and overall emissions and cost performances. Gao et al. [103] focused on the complex nature of the PVC production process and the need for continuous monitoring and fault diagnosis of critical polymerization equipment. They first improved the LMBP neural network algorithm to address its limitations. They then proposed a polymerizer device fault diagnosis algorithm based on a combination of the genetic algorithm (GA) and the improved LMBP. This GA-improved LMBP algorithm was applied to fault diagnosis in the polymerization process. By using historical industrial field data from the polymerizer, the simulation results demonstrated the effectiveness of the proposed GA-improved LMBP fault diagnosis method. Wang et al. [104] developed a real-time fault diagnosis strategy for the polymerization kettle in PVC production using a self-organizing map (SOM) neural network. The authors established a mapping between the polymerization process data and fault patterns by analyzing the production technology of the kettle equipment. They employed a particle swarm optimization (PSO) algorithm with a dynamic adjustment method for inertial weights to optimize the SOM neural network’s structural parameters. The fault pattern classification facilitated the nonlinear mapping from symptom sets to fault sets. Using industrial on-site historical data from the polymerization kettle, simulation experiments demonstrated the effectiveness of the proposed PSO-SOM fault diagnosis strategy.

Han et al. [105] presented the development of a comprehensive kinetic and heat transfer model to investigate the kinetics and predict the thermal runaway in VCM suspension polymerization. Through mapping the reactor temperature, monomer conversion, initiator moles, radical concentrations in both VC-rich and PVC-rich phases, and average molecular properties during non-isothermal processes, the study evaluates the risk of thermal runaway using S-Z (divergence) and H-J criteria, indicating that a lower jacket temperature (T_j_) and a larger heat transfer coefficient (U) can effectively delay or even prevent thermal runaway. Both the S-Z and H-J criteria demonstrate reliable predictions of thermal runaway during non-isothermal processes. To further minimize the likelihood of thermal runaway, model-based design strategies for isothermal polymerization processes were explored. Specifically, either the jacket temperature (T_j_) or the heat transfer coefficient (U) can be fine-tuned to maintain a 50 °C isothermal process, significantly reducing the risk of thermal runaway during VC suspension polymerization. Süyür, et al. [106] evaluated the pulmonary effects of exposure to PVC using high-resolution computed tomography (HRCT). A total of 104 PVC-exposed workers and 43 administrative controls participated in the study; results showed that 55% of the exposed subjects had pleural and/or parenchymal changes on HRCT. Pleural thickening was more common in the exposed group. The exposure to dust increased the risk of HRCT findings, with an odds ratio of 4.2. There was no correlation between pulmonary function tests or respiratory symptoms and HRCT findings, but HRCT changes were more common in subjects with reduced forced mid-expiratory flow. The study concluded that exposure to PVC dust, even at levels below the legal limit for respirable particulate matter, was associated with parenchymal changes and pleural thickening on HRCT.

Bottausci et al. [107] addressed the growing issue of plastic pollution by focusing on PVC, a widely used polymer in engineering infrastructures. The paper assesses the environmental impacts of PVC production to propose cleaner industrial solutions and environmentally friendly products. A LCA analysis evaluates the PVC manufacturing process, considering 1 kg of PVC granules as the functional unit. Gabi software, using three characterization methods (CML 2001, EDIP 2003, and ReCipe 1.08), facilitates the modeling. Fossil fuel depletion, climate change, and human toxicity emerge as the most significant impact categories due to crude oil extraction, emissions, and toxic substances involved in the process. The study suggests recycling and raw material alternatives to reduce the environmental impact identified in the analysis. Ye et al. [108] performed an LCA to evaluate the environmental impact of PVC production and recyclability in China. Monte Carlo simulation was used for uncertainty analysis. The main contributors to the environmental burden were found to be chlorine, carbon dioxide, and nitrogen oxides. Primary PVC production had a larger environmental impact in most categories, except for agricultural land occupation. By replacing coal-based electricity with hydropower and hybrid power, the impact on climate change could be reduced by 36.21% and 15.53%, respectively. To decrease the overall environmental impact of PVC production, it is essential to promote recycled PVC use, reduce NMVOC emissions, and increase the renewable energy ratio. Hu et al. [109] developed a pilot-scale membrane bioreactor (MBR) to treat wastewater from a PVC production site and created a simulation model based on activated sludge model no.1 (ASM1) to evaluate the MBR’s performance in terms of effluent quality, energy consumption, and reliability of operational strategies. They also proposed a modified aeration model that took into account the effect of mixed liquor suspended solids (MLSS) on oxygen transfer. The calibrated ASM1 effectively simulated NO_3_-N, NH_4_-N, and MLSS in the aerobic tank under both steady and dynamic conditions, with the pilot-scale MBR using 0.73 kWh per cubic meter of permeate production. Scenario analysis and Latin hypercube sampling were conducted to assess the influence of the sludge retention time (SRT), recirculation ratio (R), and dissolved oxygen (DO) on aeration energy demand, effluent quality, and uncertainty of model parameters. The findings suggested that the existing operational strategy had a 97% probability of achieving an effluent NH_4_-N concentration below 2 mg L^−1^.

Wu, Yu, and Chiang [110] conducted an emission assessment of a PVC plant in Taiwan using maximum achievable control technology (MACT) analysis; the assessment’s emission data were benchmarked against baseline estimates from 15 PVC plants in the USA. The research identified seven types of VCM emissions, with stripped resin being the most significant contributor (51.7%) to the average emission factor among these plants. All the emission factors met MACT emission limitations and were ranked either first or second among the 15 plants. A negative power function with a fair correlation (R^2^ = 0.73) was observed when emissions were compared to each plant production capacity. Mei et al. (2019) used coconut-activated carbon (CAC) to address two environmental issues: dyeing wastewater pollution and mercury contamination from PVC production. This catalyst demonstrated excellent performance in acetylene hydrochlorination, an essential reaction for the PVC industry. They performed molecular dynamics simulations, and density functional theory (DFT) calculations to investigate the structural and electronic properties of the N-doped CAC catalysts, the nitrogen doping process, and the catalytic mechanism of different N species for acetylene hydrochlorination.

Li et al. [111] evaluated the contributions of various chlorinated paraffins (CPs) sources in the environment, including CP products, metalworking, and PVC production, focusing on the effects of CP-related emission industries on regional atmospheric characteristics of CPs. The researchers collected 60 passive air samples from five cities in Henan Province, China, which had serious CP pollution and different CP-related emission industry structures, finding higher concentrations of short chain CPs (SCCPs) and medium chain CPs (MCCPs) than in most previous reports. The study found that the local CP-related emission industrial structure had a greater impact on MCCPs pollution than SCCPs. Notably, the contribution of the metalworking industry to CP pollution was more significant than that of the PVC production industry and CP products industry.

The production of polyvinyl chloride (PVC) involves the use of vinyl chloride monomer (VCM) and various additives, which can lead to the emission of volatile organic compounds (VOCs) [112]. The PVC production process can result in the release of harmful substances such as carcinogenic vinyl chloride [71]. Additives used in PVC production, such as waxes, stabilizers, plasticizers, flame retardants, and pigments [113,114], can also contribute to VOC emissions [115,116]. These VOCs can contaminate the air and drinking water supplies, contributing to environmental degradation and climate change.

Studies have shown that exposure to these VOCs can lead to health issues such as respiratory problems [114]. For instance, it has been revealed that PVC production releases hundreds of thousands of pounds of carcinogenic vinyl chloride into the air every year [117,118]. Another study commissioned by the European Commission in 2020 aimed to identify and describe uncertainties about PVC production, recovery, and end-of-life treatment from a chemicals perspective [71]. It identified the presence of VOCs 2-ethylhexanol and triacetin when using PVC-based clingfilms; the substances were considered as a “non-intentionally added substance” [119]. It was also found that most VOCs in indoor microenvironments originated from the melting extrusion process when recycling PVC, especially oxygenated VOC mainly conformed by cyclopentanone, n-butanol, methyl methacrylate, and a small fraction of chlorinated VOC; these results implied that PVC tended to be oxidized by the heat, implying dehydrochlorination at a low temperature leading to the formation of HCl as well as polyene free radicals [120]. These studies highlight the need for further research and regulation to mitigate the environmental and health impacts of PVC production in the world.

## 5. Conclusions

Bibliometric and co-occurrence studies have allowed us to identify trends in research interests, research gaps and future directions and challenges regarding process system engineering applied to PVC production while co-occurrence analysis identified five distinct word clusters related to PVC and simulation. The relevant areas of interest for further investigation are mathematics and statistics applied to polymer chemistry, separation phenomena, and polymer production and applications. The analysis of research trends with time revealed that there was a strong focus on Monte Carlo methods and numerical simulation, contributing to a deeper understanding of PVC’s properties and behavior. Subsequently, research shifted towards biomedical applications and interactions with biological systems. More recently, the focus has expanded to encompass broader studies of plastics and microplastics, reflecting growing concerns about environmental impact and the need for sustainable solutions. Alongside this, the development of new algorithms for simulation methods has become increasingly important to enhance accuracy and efficiency across various applications.

Regarding bibliometric study, studies showed that PSE gives the tools for improvement of traditional PVC production processes by employing advanced process engineering techniques; for instance, the modelling and simulation of continuous polymerization processes, micro-reactor systems, and the use of novel catalysts to enhance conversion rates, product quality, and energy efficiency. As the PVC production process is energy-intensive and most of the energy consumed is derived from fossil fuels, it is important to minimize the environmental impact and reduce greenhouse gas emissions. Incorporating renewable energy sources such as solar, wind, or geothermal energy into PVC production processes is essential with a decarbonization strategy. This could involve adopting novel technologies like solar-driven steam generation, waste heat recovery systems, or the use of renewable electricity in process operations. Energy management systems and advanced process control strategies can be implemented to optimize energy consumption and integrate renewables effectively. From the economic point of view, developing a circular economy approach for PVC production and waste management will require innovations in material design, recycling technologies, and the development of effective end-of-life strategies for PVC products which can be evaluated via process simulation. Research into PVC materials with enhanced recyclability and/or biodegradability can help reduce the environmental impact of PVC waste. Process integration techniques, such as pinch analysis, mass integration, energy integration or property integration can be employed to minimize energy consumption and reduce waste generation in PVC production processes. Finally, it is crucial to assess the sustainability of PVC production processes by employing methods such as life cycle assessment (LCA), techno-economic analysis and techno-economic resilience evaluation, environmental risk assessment (ERA), social impact evaluation, exergy analysis, and safety evaluation, among others. These evaluations will help identify potential environmental and social impacts, assess economic viability and response to changes in the economic environment of the process, and gauge the overall sustainability performance of PVC production technologies. They can also guide the development of new technologies and process improvements in alignment with environmental, energetic, social, and economic objectives.

Process manufacturing is expected to be influenced by several key trends such as: Digitalization of distributed control systems (DCSs), modular approach, integration of IT components, and membrane processes. DCSs are at the heart of process manufacturing operations and have evolved to focus on digitalization. The use of pre-made and pre-tested automation software modules will allow companies to use DCSs more effectively. The future DCSs are expected to become more flexible, allowing them to add new functions easily and adapt as their users’ needs change. A modular approach allows parts of the system to be changed without replacing the entire system and could be achieved through a radical change in the architecture of the DCS, separating the control system core functions from the less critical features. Integration of IT components: The future will see an extended automation environment that helps better collaboration between people, systems, and equipment. This is achieved through building an open and secure environment that can integrate IT components from the field up to the enterprise level. The use of edge and cloud computing technology will help companies achieve the benefits of IIoT-enabled sensors. Companies can collect and analyze huge quantities of data while sharing them across the organization. Membrane processes are key technologies for industrial separations and are expected to play an important role in future sustainable production systems. The combination of materials science and process engineering has historically always been an essential condition for the development of new applications for membranes.

Applying the prior concepts, PSE can be implemented in biopharmaceuticals and biotechnology, food and beverages production, renewable energy, chemical and petrochemical industries (like PVC), environmental engineering, pharmaceutical manufacturing, advanced materials, electronics and semiconductors manufacturing, mining and mineral processing, and water treatment and desalination. The future of PSE in these fields will likely involve greater integration of advanced data analytics, artificial intelligence, machine learning, and automation to enhance decision-making and process control. Additionally, sustainability and environmental considerations will continue to drive innovation in process design and optimization, with an emphasis on reducing waste, energy consumption, and environmental impact. As industries evolve and adopt new technologies, PSE will remain a key driver of efficiency, sustainability, and competitiveness in process manufacturing.

## Figures and Tables

**Table 1:** Comparative summary between suspension, emulsion and intrinsic polymerization methods.

Suspension	Emulsion	Intrinsic (Bulk)
Suspends hydrophobic monomers in an aqueous phase. Requires energy-intensive mechanical agitation. Heat transfer is less efficient due to solids present. The monomers are in the liquid phase and the polymer material exists as a sphere suspended in the liquid [56]. The degree of polymerization can be obtained by modifying the process conditions measuring the molecular weight of the polymer by solution viscosity [57]. Control over particle size and distribution is challenging. Used for particles, microspheres, bead polymer production, and copolymerization [58]. Used for particles, microspheres, and bead polymer production. Control over particle size and distribution is challenging [59].	Disperses monomers in micelles within an aqueous phase. Moderate agitation needed to maintain emulsion. Heat transfer can be more efficient due to liquid phase [60]. Offers versatility for a wide range of monomers, such as latex production for paints, though it demands surfactants and careful pH control [61]. Suitable for copolymerization [62]. Emulsifiers and stabilizers may be needed [63,64]. Allows control over droplet size and distribution.	Occurs in neat monomers without a dispersing medium. Can be carried out in a single-phase system [65]. No external agitation required. Efficient heat transfer within a homogeneous phase [66]. Employed for monomers that are liquid or can be melted, like polyethylene and polypropylene. Offers a simplified process but without the versatility of emulsion polymerization [57]. Suitable for making linear or branched polymers, and copolymerization. Fewer waste byproducts. No particle size control as it occurs in a homogeneous phase [67].

## Data Availability

The data will be available upon reasonable request to the corresponding author (Á.D.G.-D.).

## Nomenclature

AFM	Atomic Force Microscopy
BP	Back Propagation
CAC	Coconut Activated Carbon
CiD	Continuous Initiator Dosing
CMRP	Cobalt-Mediated Radical Polymerization
CPs	Chlorinated Paraffins
CS	Cuckoo Search
D-FNN	Dynamic Fuzzy Neural Network
DEA	Data Envelopment Analysis
DFT	Density Functional Theory
DO	Dissolved Oxygen
EDX	Energy-Dispersive X-Ray
E-PVC	Emulsion Polyvinyl Chloride
ERA	Environmental Risk Assessment
FT-IR	Fourier Transform Infrared Spectroscopy
GA	Genetic Algorithm
HCl	Hydrogen Chloride
HRCT	High-Resolution Computed Tomography
LCA	Life Cycle Assessment
LMBP	Least Mean Square Algorithm Back Propagation
MACT	Maximum Achievable Control Technology
MBS	Methyl Methacrylate–Butadiene–Styrene
MBR	Membrane Bioreactor
MCCPs	Medium Chain Cps
mGPDP	multi-modal Gaussian Process Dynamic Programming
MILP	Mixed-Integer Linear Program
MINLP	Mixed-Integer Nonlinear Programming
MIS	Copolymers of Isoprene (Is), Styrene (St), And Methyl Methacrylate (MMA) Monomers
MLSS	Mixed Liquor Suspended Solids
M_w_	Molecular Weight
nano-CaCO_3_	nanometer Calcium Carbonate
NMR	Non-Methane Volatile Organic Compounds
PSA	Process System Engineering
PSD	Particle Size Distribution
PSO	Particle Swarm Optimization
PVC	Poly(Vinyl Chloride)
R	Recirculation Ratio
RBF	Radial Basis Function
RS	Rough Set
SEM	Scanning Electron Microscopy
SCCPs	Short Chain Cps
SDFM	Solution-Diffusion-Film Model
SOM	Self-Organizing Map
s-PVC	Suspension-PVC
SRT	Sludge Retention Time
SVM	Support Vector Machine
TDI	Toluene Di-Isocyanate
TEM	Transmission Electron Microscopy
T_g_	Glass Transition Temperature
T_j_	Jacket Temperature
TOC	Theory of Constraints
tTDI	Toluene Di-Isocyanate Trimer
Vac	Vinyl Acetate
VCM	Vinyl Chloride Monomer
